# Response to: "Letter to the Editor– Observation on the article titled “Vaccine-induced thrombotic thrombocytopenia (VITT): first report from India”"

**DOI:** 10.1186/s12959-023-00474-7

**Published:** 2023-04-24

**Authors:** Christy V. John, Rajesh Kumar, Anil Kumar Sivan, Sangeetha Jithin, Rojin Abraham, Chepsy C. Philip

**Affiliations:** Believers Church Medical College Hospital, Thiruvalla, Kerala 689103 India

We appreciate the interest and comments of Kotwal, J and Balraam, V V K in our article detailing the diagnosis and treatment of vaccine-induced thrombotic thrombocytopenia ( VITT) [1].

We have been careful and made efforts to qualify our case report on the absence of prior reports from India based on the data submitted to the AEFI committee in India and also on our search in Pubmed and Google using the search terms “VITT” and “ India” [2]. We repeated the search both at submission as a preprint and to the journal [3].

Our primary goal was to share our experience in considering VITT and to initiate treatment while awaiting confirmation of diagnosis, even while working within the constraints of resources. The authors would concur that we had done a careful search and would not have identified manuscripts under consideration which are yet to be published. We are unequivocal in acknowledging the limitations of our best efforts, which we have qualified in our manuscript, and we agree that VITT is a potentially devastating but treatable condition that benefits from a high index of suspicion [4].
